# Human Dignity as a Component of a Long-Lasting and Widespread Conceptual Construct

**DOI:** 10.1007/s11673-014-9512-9

**Published:** 2014-04-22

**Authors:** Bernard Baertschi

**Affiliations:** Institute for Ethics, History, and the Humanities, University of Geneva, CMU 1, rue Michel-Servet 1211, Geneva 4, Switzerland

**Keywords:** Dignity, Intrinsic value, Person, Moral status, Slavery, Torture

## Abstract

For some decades, the concept of human dignity has been widely discussed in bioethical literature. Some authors think that this concept is central to questions of respect for human beings, whereas others are very critical of it. It should be noted that, in these debates, dignity is one component of a long-lasting and widespread conceptual construct used to support a stance on the ethical question of the moral status of an action or being. This construct has been used from Modernity onward to condemn slavery and torture as violations of human dignity. In spelling it out, we can come to a better understanding of what “dignity” means and become aware that there exists a quite useful place for this notion in our ethical thought, albeit a modest one.

## Introduction

In recent decades, the invocation of *human dignity* has become increasingly frequent in bioethical debates and has also come under growing criticism. Some authors deplore the degree to which it is now prevalent and fear that it could become an irremediably confused concept. Consequently, papers have appeared with denigrating titles such as “Dignity Is a Useless Concept” (Macklin 2003) or even “The Stupidity of Dignity” (Pinker 2008), while others have called for an “Undignified Bioethics” (Cochrane 2010, 234)—that is, for a bioethics without the language of dignity. If these views are correct, it would be a big problem for all texts—moral and legal, national and international—that place the concept of human dignity at their center. However, to fully measure the problem, the debate on the concept’s utility must be supplemented by efforts toward a better understanding of it. The present paper focuses mainly on this task. It is not the first to tackle this issue. For instance, Rieke van der Graaf and Johannes van Delden (2009) published a paper on the history of dignity, with the aim of clarifying the concept and justifying a moderate and reasonable use of it in contemporary bioethics. This present paper will focus more on the conceptual roots of dignity.

To understand adequately the conceptual structure of “dignity” and its conceptual links to other important notions of ethics (such as “intrinsic value,” “moral status,” and “personhood”), it is nevertheless useful to consider some historical aspects.^1^ This is the focus of the first section. It will then be possible to gain a better grasp of contemporary uses of “dignity” (second section) and of the differences regarding the contexts of its use in Europe and the United States (third section). In the last section, I will examine the usefulness of the concept and propose what I deem to be an appropriate role for it in contemporary ethical debates.

Before addressing the subject of human dignity, it is important to make some distinctions in order to avoid misunderstandings. “Dignity” is restricted neither to *human dignity*, nor to ethics. In everyday language, we speak of dignity in the social domain (sometimes called “dignity of merit”). In some languages, such as French and German, “worthy” is translated by adjectives derived from “dignity”: *digne* comes from *dignité*, *würdig* from *Würde*. In ethical matters, “dignity” (and “indignity”) is used in three different contexts, with three different meanings. Dignity is sometimes linked to *what we do* (we respect our own dignity when we act rightly, we lose it when we act wrongly), sometimes to *what we suffer* (when we are not treated as we deserve to be treated, we can experience a feeling of indignity), and sometimes to *what we are*.^2^ I am specifically concerned with the third type of dignity. Because it is tied to what we are—i.e., to our nature—it cannot be lost. Kant was already aware of this when he stated: “I am not entitled to refuse, even to the vicious, all consideration in his capacity as a man, this last being inalienable, although the other make himself unworthy of it,” because “humanity is itself a dignity” (Kant 1886, 170–171).^3^ It is important not to confuse the three meanings. For instance, in the debate surrounding cloning, the claim that this process goes against dignity is thought by some to be problematic, because it implies in their minds that a child procreated by cloning would be deprived of dignity. This manner of thinking endures, argues Pinker (2008, 5). Of course, the thesis that cloning is against dignity is problematic, but not for the reason given by those authors who confuse *human* dignity (that associated with *what we are*) with *action* dignity. Cloning may be or may not be a violation of human dignity through action indignity, but it cannot destroy human dignity.

The same can be said for torture and all violations of human dignity. What can be violated cannot be lost “as long as the persons exist, even in case of extreme bodily and cognitive deterioration,” add Chris Gastmans and Jan De Lepeleire. Therefore, “loss of dignity cannot be used as an argument for euthanasia in persons with severe dementia” (Gastmans and De Lepeleire 2010, 84). Unfortunately, this last claim equivocates: loss of *human* dignity cannot, but loss of *personal* dignity could perhaps.

## Roots and Conceptual Content

In this section, I will not dwell on the details of textual interpretation. My aim is to shed light on conceptual structures and contents that help us to understand the current use of human dignity, because they have remained rather constant for centuries and are typical of a Western understanding of morality when it pertains to the moral status of human beings. Sometimes, attention to detail can hinder us from seeing general orientations. Moreover, I want to stress that the conceptual structures analyzed here are not part of the opposition between deontologists and consequentialists, even if the former are more prone to use the concept of human dignity.

When we ask: “Where does the concept of *dignity* comes from?” philosophically minded people answer: “From the philosophy of Kant.” It is true that the German philosopher uses this concept frequently and that it occupies a central place in his moral thought when he addresses the question of what we now call “the moral status” of human beings. It is well known that Kant contrasts dignity with price:

“In the realm of ends everything has either a *price* or an *intrinsic value* [*Würde*]. Anything with a price can be replaced by something else as its equivalent, whereas anything that is above all price and therefore admits of no equivalent has intrinsic value (Kant 2008, 33, *emphasis original*).”

Bennett translates “dignity” by “intrinsic value,” because, as he says in a footnote: “At the end of the next paragraph Kant explicitly equates those two meanings, when he speaks of ‘intrinsic value’ (i.e. dignity) [*einen innern Wert, d.i. Würde*]” (see Kant 2008, 33).^4^ For morality and law, says Kant, there exists only two kinds of beings—and consequently two kinds of moral status—human beings (or persons) and things. Things can be bought or sold, therefore they have a price and are replaceable by other things of the same price; persons cannot be bought or sold, therefore they have no price and are not replaceable. They, and only they, have dignity. By their nature, things are entities that can be instrumentalized; persons should not, as the second formula of the categorical imperative states: “Act in such a way as to treat humanity, whether in your own person or in that of anyone else, always as an end and never merely as a means” (Kant 2008, 29). Persons should not be used as mere means to an end, that is, instrumentalized.

People who are not philosophically minded generally do not know the role of Kant, but do feel his influence: The prohibition of instrumentalization is everywhere in debates involving human dignity. Philosophically minded people are aware of this influence, but few of them know that Kant’s concept of dignity has a long history. It was already in force in the 13th century. We read in Aquinas’ *Commentary on the Sentences* this passage: “Dignity means the goodness something possesses because of itself, utility its goodness because of another” [*dignitas significat bonitatem alicujus propter seipsum, utilitas vero propter aliud*] (Aquinas 2012, lib. 3, d. 35, q. 1, a. 4, q. 1, c). In contemporary words, dignity means the intrinsic value of something, utility its instrumental value. Kant agrees, but there is a big difference between the two authors on this point: For Kant, dignity is a property of *beings*, whereas for Aquinas, it is a property of everything that possesses an intrinsic value. For the latter, some activities possess such a value; to illustrate the distinction, he mentions the greater dignity of contemplation in comparison with active life.

Coupled with the metaphysical thesis that *being* and *goodness* are coextensive, Aquinas’ position implies that every being has a dignity and not only human beings. He states this explicitly regarding beings that are worthier (*dignior*) than human beings, angels, and God: “The dignity of the divine nature exceeds every other dignity” (Aquinas 1947, Ia, q. 29, a. 3, ad 2). However, in principle, dignity could be attributed to entities that are less worthy than human beings, such as animals, because each nature possesses its own dignity. Aquinas uses the expression *dignior*—“worthier”—modeling a scale of beings in terms of value or dignity. Kant is far from this metaphysical approach, reserving dignity for human beings.

The formal conceptual content of dignity (dignity as intrinsic value) is, nevertheless, constant from Aquinas to Kant. This much is obvious from the texts and from their opposition of dignity to utility and instrumentalization. Indeed, the analogy goes deeper: For both authors, dignity has the same place in the ethical landscape. Let us consider this more precisely.

As we see from the writings of Aquinas and Kant, dignity is rooted in intrinsic value in the sense that it *is* intrinsic value. But from where does this value come? The intrinsic value of an entity comes from its intrinsic properties (because of *itself,* said Aquinas). With regard to human dignity, the relevant intrinsic property is *reason* in one form or another. Aquinas speaks generally of “intellectual nature”: “The nature which *person* includes in its definition is of all natures the most exalted [*est omnium naturarum dignissima*], to wit that nature which is intellectual in regard to its genus. Likewise the mode of existence signified by the word *person* is most exalted [*dignissimus*], namely that a thing exists by itself” (Aquinas 1952, q. 9, a. 3, *emphasis original*).

Kant very often mentions autonomy, but self-consciousness too, particularly in an illuminating passage:

The fact that man is aware of an ego-concept raises him infinitely above all other creatures living on earth. Because of this, he is a person. … He is a being who, by reason of his preeminence and dignity, is wholly different from things, such as the irrational animals, which he can master and rule at will (Kant 1978, 9).

As it appears in this last quotation, if reason is the relevant property, it is because it is characteristic of human beings, as opposed to non-rational sensibility or sentience, which is characteristic of animals. What counts as dignity is, therefore, not any intrinsic property, but intrinsic properties that are characteristic of beings, properties that belong to their essence. Sentience is an intrinsic property of human beings, but not one that is characteristic of human beings. Therefore it is not suitable for human dignity. Recently, Rieke van der Graaf and Johannes van Delden have stressed that human “dignity is based on essential human characteristics” (Van der Graaf and van Delden 2009, 158), while Adam Schulman links dignity with “the essential and inviolable core of our *humanity*” (Schulman 2008, 17, *emphasis original*).^5^

Reason is the basis of dignity. If we tried to explain more precisely their relation, we should say that dignity supervenes on reason.^6^ However, human beings possess several essential or distinctive intrinsic properties. Why does reason hold such a privileged status? Aquinas and Kant would give a different answer to this question, because it is a metaphysical one and their metaphysics differs widely. Yet what is common to them is the widely shared thesis that *rationality is what distinguishes human beings from animals*.

Three more examples of this. The French poet Pierre Ronsard writes in the 16th century that only reason separates human beings and beasts and that reason is a faculty lodged in the brain, having control over the body.^7^ One century later, the philosopher Blaise Pascal states: “Man is obviously made to think. It is his whole dignity and his whole merit; and his whole duty is to think as he ought” (Pascal 1660, 33). At the same time, Samuel von Pufendorf, an author well acquainted with scholastic thought and who has influenced Kant’s work, asserted that “the word *man* is thought to contain a certain dignity,” allowing us to respond to someone disrespecting us that we are men, not dogs (Von Pufendorf 1964, 47, *emphasis original*).

Nowadays, this kind of anthropocentrism is often doubted, because we have discovered that many rational faculties are more or less present in some animals—but our deep valuation of rationality has not vanished. It is notably visible in our general stance toward the loss of rational faculties: Such loss is generally considered the worst thing that can affect us. For most of us, reason remains internally linked with high value, in such a way that we seldom ask for a justification of this link. It is a kind of moral intuition.

Amongst the intrinsic properties that characterize human beings, reason is the basis of dignity, say Aquinas, Kant, and the tradition they exemplify. To be precise, reason is not conceived as a characteristic of a human being, but of a *person*. Western philosophers from Boethius onwards have proposed that a person is an individual endowed with reason.^8^ Aquinas and Kant tie personhood to dignity and to reason. Aquinas states:

In a more special and perfect way, the particular and the individual are found in the rational substances which have dominion over their own actions; and which are not only made to act, like others; but which can act of themselves. … Therefore also the individuals of the rational nature have a special name even among other substances; and this name is “person” (1947, Ia, q. 29, a. 1).

Nowadays, authors such as Tristram Engelhardt emphasize the same conceptual structure, even if he does not use the concept of dignity in this context: “What distinguishes persons is their capacity to be self-conscious, rational, and concerned with worthiness of blame and praise. The possibility of such entities grounds the possibility of the moral community” (Engelhardt 1986, 107).

He is not alone in this way of thinking. The *Universal Declaration of Human Rights* also links human dignity to reason, but there is an ambiguous understanding of “person” and “human being,” as we read in Article 1: “All human beings are born free and equal in dignity and rights. They are endowed with reason and conscience.” Strictly speaking, this is false: Every human being is not endowed with reason and conscience, even if we understand reason as a potentiality (think of anencephalic babies). Contrariwise, it is by definition true that every person is endowed with reason and conscience. This ambiguity is rather widespread; even Kant speaks sometimes of “personhood,” sometimes of “humanity.” It is the source of many problems, particularly the question of the moral status of so-called marginal human beings, i.e. human beings who are not persons in the sense defined above (embryos, anencephalic babies, PVS patients, etc.).

We can now summarize the formal structure we have highlighted in the following manner: *Person* is the name of a *moral status*. What is distinctive about this status is the possession of *reason*, an intrinsic property that gives its bearer an intrinsic value or *dignity*.

Some remarks are still in order.

First, from Kant onwards, the tendency has been to reserve dignity exclusively for human beings. Earlier, this was not the case: As we have seen, Aquinas extends dignity—and personhood—to angels and God. Dignity, however, was not attributed to non-rational beings, even if its definition could have allowed it, since intrinsic value is not exemplified in rationality alone. As Lennart Nordenfelt states, there is of course a good conceptual reason not to attribute dignity to lower beings: “Dignity refers to a special dimension of value” (2004, 70). That is, to a high place on the scale of values, a scale where human beings have traditionally occupied the highest place in the realm of natural beings. If we abandon this anthropocentrist view, which is an increasingly common position to adopt, dignity could be attributed to beings we value, such as apes, whales, or even native forests. As we will see later, the Swiss Constitution exemplifies this trend.

Second, the fact that dignity refers to intrinsic value and is attributed to non-human beings (i.e., super-human ones) clearly shows that, in our moral tradition, “person” and not “human being” is the right designation for the relevant moral status of beings like us. More precisely, “human being” is not the name of a moral status. It is important to emphasize this point, because some authors—notably Leon Kass—have used the concept of dignity to oppose the personhood account, which deprives marginal humans of full moral status (Ashcroft 2005). As Kass says: “The account of human dignity we badly need in bioethics goes beyond the said dignity of ‘persons’ to embrace the worthiness of embodied human life” (Kass 2008, 313). Many international declarations on dignity also focus on humanity rather than personhood (Cutas 2005). What Kass and others say may be right, but it is not in keeping with the traditional concept of dignity. An alternative position, which claims that “[t]he dignity of ‘being a person’ should not depend on whether one has or does not have certain capacities (e.g. intellectual capacities)” (Gastmans and De Lepeleire 2010, 81), does not correspond with the dignity tradition either. Conversely, the conceptions of the deontologist Tristram Engelhardt and of the utilitarian Peter Singer are in tune with the traditional concept of dignity, even if they insist on personhood rather than dignity.

Third, many contemporary authors assert that human dignity is rooted in the belief that human beings have been created in God’s image (Kraynak 2008). Even if such an idea is easily derived from the concept of person, since a human person is endowed with the same reason as God, the conceptual structure I have underlined is independent of any religious thesis. As we shall see later, it is also independent of any conservative thesis.

Fourth, the conceptual status of “person” is not clear. For Engelhardt and most *personists*, being a person is an individual predicate. An individual is a person if and only if he or she—as an individual—possesses rational powers. For other authors, being a person is a natural kind predicate. Therefore, an individual is a person if and only if he or she belongs to a kind whose standard members possess rational powers. Daniel Sulmasy defends such a theory when he argues: “It is not the expression of rationality that makes us human, but our belonging to a kind that is capable of rationality that makes us human” (Sulmasy 2008 478). Kant was very probably an individualist, but it is not clear whether Aquinas was. Notice that a natural kind conception allows us to avoid the marginal human beings’ problem, but at a very high price for many individualists, since it places high barriers in the way of abortion or embryo research, for example.^9^

Last, a metaethical remark. The conceptual structure does not have any privileged relationship with moral realism. It states that intrinsic value supervenes from intrinsic properties, but remains silent on the nature of this relationship. It can be read in a realist spirit, but in an antirealist spirit too, if, for instance, we understand it in the following manner: The intrinsic value of *x* is projected on *x* on the basis of its intrinsic properties.

## Current Ethical Content

The conceptual content of dignity has been held constant for a long time, and the conceptual structure it weaves with moral status, personhood, and reason has also remained essentially the same throughout the centuries. The first challenge to this structure was the birth of utilitarianism and its objections against the weight and the value of reason in ethics. In terms of the formal structure itself, however, the challenge has not been crucial: For the Benthamists, sentience simply replaced reason as the distinctive property of moral status, and other utilitarians have not abandoned the concept of person. Further dissent between moral philosophers concerns the opposition we find between monists and pluralists. I use these terms with the following meaning: A monistic conception of moral status argues that there exists only one moral status (either you have a moral status or you have no moral status); utilitarians and Kant are monists. In comparison, a pluralistic conception states that there exist several kinds of moral status, depending on different properties; for example, reason for persons, sentience for animals, life for plants. Nevertheless, for both sides of this debate, the formal structure remains the same.

Historically, the first full occurrence of this construct of which I am aware is Aquinas’ view, but parts of it are found much earlier. The contrast between human beings and animals understood as a contrast between reason and sentience (affective life) is well articulated in Aristotle’s philosophy. We also find a moral consideration against bestiality in the name of human dignity in Cicero’s texts:

One’s physical comforts and wants should be ordered according to the demands of health and strength, not according to the calls of pleasure. And if we will only bear in mind the superiority and dignity of our nature, we shall realize how wrong it is to abandon ourselves to excess and to live in luxury and voluptuousness, and how right it is to live in thrift, self-denial, simplicity, and sobriety. … [We are] all alike endowed with reason and with that superiority which lifts us above the brute (Cicero 1913, 109).

This view persisted in the middle ages (Dale 1977) and beyond, as we have seen with Ronsard, Pascal, Pufendorf, and Kant.

I have argued that this conceptual structure is still with us. But what about its ethical content? From the time of Aquinas, the moral landscape has changed. Questions pertaining to the good life of the agent (the person) have largely been replaced by questions concerning right actions toward other persons. Nevertheless, questions related to moral status and dignity remain. The moral status of an entity implies how we ought to treat it. As Mary Anne Warren stated: “If an entity has moral status, then we may not treat it in just any way we please” (Warren 1997, 3). In Aquinas’ time, this demand was spelled out in a theory of the various virtues we should cultivate, so there is an inescapable reference to duties toward oneself in this approach to morality (hence the condemnation of bestiality). In modern times, this reference has been largely downplayed in favor of duties toward other persons.

Injunctions to respect dignity have thus taken an impersonal or an other-directed turn. In Kant’s writings, we observe the two trends, with a stress on duties toward oneself: “Ethics gives no title to vice on account of its harmlessness; for the dishonour (i.e., to be an object of ethical disdain) it entails, accompanies the liar like his shadow. … A lie is the abandonment, and, as it were, the annihilation, of the dignity of a man” (Kant 1978, 148).

In a contemporary context, liars are not condemned in this manner, but the question of respecting dignity in oneself has not totally disappeared, even if it is contested (Cutas 2005). In our liberal tradition, however, dignity is mainly concerned with what we ought to do to other human beings.

What then does it now mean to respect human dignity? The answer is usually expressed in two bans: a ban on instrumentalization and a ban on degrading treatments or humiliations.

These two bans are conspicuous in internationally important texts. For example, in the preamble of the *Convention for the Protection of Human Rights and Dignity of the Human Being with Regard to the Application of Biology and Medicine* (Oviedo Convention), we read that the European Council is “conscious that the misuse of biology and medicine may lead to acts endangering human dignity,” in the sense that biotechnologies could treat human beings in a manner not appropriate for the being they are. In the *Universal Declaration on the Human Genome and Human Rights* proclaimed by UNESCO, we find the same lesson, for example, in Article 2: “Dignity makes it imperative not to reduce individuals to their genetic characteristics and to respect their uniqueness and diversity.” Medicine and biology could reduce human beings to the status of objects for experiment or could consider them as completely determined by their genome, thus treating them as non-persons (Beyleveld and Brownsword 2002).

The ban on instrumentalization is the most voiced of the two bans. As we know, it appeared in Kant’s second formulation of the categorical imperative. However, it is in one sense less fundamental than the ban on degrading treatments, because instrumentalization is only one kind of degrading treatment: It degrades a human being to the status of a thing. Nevertheless, if the two bans are kept separate, it is because each points to a different paradigm: The paradigm of instrumentalization is slavery, whereas the paradigm of degrading treatment is torture. Notice that if torture is such a paradigm, it is not because of the pain inflicted but because pain has the effect of inducing emotions and behaviors that are in contradiction to what the victim wants to feel and to be as a free and rational person. Historically, slavery and torture are perhaps the two main domains where human beings have been (and unfortunately still are) treated with total disrespect. Thus, it is not surprising that they are mentioned so often in international legal texts.

Torture is clearly bad; instrumentalization is bad, too, but is often much more difficult to detect, which is probably why it has attracted more attention. A reinforcing factor is that instrumentalization has an internal link with liberty or autonomy, a rational property of human beings that is so important for contemporary ethics. The link can be articulated in seven propositions:

1. A person is a rational being.
2. Rationality includes the freedom and power to conduct one’s life; i.e., to determine one’s goals (autonomy).
3. A behavior that does not respect a person’s autonomy does not respect that person.
4. When we instrumentalize a person, we substitute or impose our own goals.
5. Therefore, instrumentalization amounts to a lack of respect for a person.
6. To have no respect for someone as a person amounts to a violation of dignity.
7. Therefore, to instrumentalize a person amounts to a violation of his or her dignity.

*This argument shows clearly that contemporary uses of dignity are rooted in its traditional meaning*: To instrumentalize or degrade a human being manifests a lack of respect for that being; i.e., for his or her intrinsic value. It amounts to treating an individual like—literally—an *Untermensch*. Another striking illustration that the contemporary use of dignity is rooted in its traditional meaning is UNESCO’s *Bioethics Core Curriculum*, where we read that human dignity is “an *intrinsic value* of the *person* capable (at least potentially or as a member of natural kind) of *reflection*, sensitivity, verbal communication, free choice, self-determination in conduct and creativity” (UNESCO 2008, 19, *emphasis added*).

Notice that this seven-steps argument should be made more specific in order to answer some objections. Remembering Kant, we could highlight that a liar violates not only his or her own dignity but also the dignity of his or her victim, since in lying he or she manipulates his or her victims and, in a certain sense, imposes his or her own goals on the victim.^10^ However, to repeat a well-known argument, we have a duty to lie to a Nazi who is looking for a resistance fighter hiding in our cellar. Does it mean that we have a duty to violate the Nazi’s dignity? Kant answered negatively: We never have a duty to lie, because we always ought to act under the supposition that the person in front of us will behave morally. However, (almost) nobody follows him in his opinion, because we usually think that it is not morally responsible to act as if evil persons did not exist. How then to answer the question? Several paths can be contemplated, but in my mind the most promising is to consider that imposing foreign goals on a person does not consist in a violation of his or her dignity when his or her own goals are not rationally pursued or morally permissible. Otherwise, punishment and even education would consist in violations of human dignity.

## Contemporary Contexts of Use

Understood in this way, dignity seems to be quite an honorable concept. Why, then, all this fuss about it? In my opinion, it comes primarily from some of its uses. But the contexts in which dignity is now referred are manifold and do not necessarily pose any special problem.

First, as already mentioned, there is the *Universal Declaration of Human Rights* of 1949. The previous declaration of 1789 did not mention dignity, but only the equality of rights. Several international texts refer explicitly to that *Universal Declaration*, especially in the domain of biotechnology. National legal texts mention dignity, some in their constitution (especially Germany and Switzerland) and others in various legal documents (for example, France and Sweden). This use, inspired by the *Universal Declaration*, is neutral in the sense that it does not correspond to any partisan doctrine: It is not conservative or progressive, rightist or leftist, religious or secular, even if it suffers from the ambiguity between person and human being. As a *legal* concept, it has no specific content: As several constitutionalists have emphasized, its content is provided by different fundamental rights, especially autonomy and personal liberty. In Switzerland, for example, lawyers believe that human dignity is directly tied to personal liberty, that it is an objective principle that must be protected and respected in the whole of the legal order but which cannot be called upon unless liberty or another fundamental right is in jeopardy (Auer, Malinverni, and Hottelier, 2000).^11^ Even so, for most lawyers it is not a “useless concept” because it signifies a commitment to the particular value of human beings. As Roberto Andorno notes:

It is indeed difficult, if not impossible, to provide a justification of human rights without making some reference, at least implicitly, to the idea of human dignity. This notion is usually associated with supreme importance, fundamental value and inviolability of the human person (Andorno 2002, 960).

If this first use is widespread in Europe, a second is characteristic of many debates in the United States. Here, dignity is a partisan concept, claimed by conservatives. Dignity underwent a renewal of fame when Leon Kass was president of the Council on Bioethics, and it now flourishes in American conservative circles. It is against this use that Steven Pinker has spoken of the *stupidity* of dignity. The problem with the conservative use is, as Ruth Macklin states, that “appeals to dignity continue to abound … without any attempt to define or analyze the concept” (Macklin 2006, 37), as if dignity were a primitive concept, a verbal expression for the perception of an unanalyzable value. Kass, however, goes into some detail. He takes on the formal and traditional content of dignity when he speaks of “the dignity or worth or standing of the human creature” that “reveals that elusive core of our humanity, those special qualities that makes us more than beasts yet less than gods” (2005, 240). He also puts substantial content in it by providing a list of goods relevant to dignity. *Unfortunately, what is lacking is a justification of the inclusion of the items in the list*, something that actually belongs to the core of the bioethicist’s task and is necessary.

This debate is not absent in Europe: The Catholic Church and fundamentalist Protestant circles have objected to artificial procreation and to experimentation on embryos—and even to biotechnology in general—in the name of human dignity. But they are not alone, and we do not observe a left–right divide on these topics.

The debate surrounding euthanasia and assisted suicide has produced a third context of the concept’s use (though it may have been the first chronologically). Both sides have asked for a dignified death—i.e., a death in conformity with our humanity (or our status as persons). However, dignity has received two different contents in this debate, depending on the side embraced by the user.

Finally, recent debates in some European countries have demonstrated a tendency to extend the scope of dignity to non-human animals (and even to all living things, as in the Swiss Constitution). This extension is in agreement with the conceptual content of dignity (if non-human beings have an intrinsic value, then they have dignity) and could be in tune with our contemporary sensibility to values, but is rather difficult to articulate with human dignity because human dignity is indicative of rights, whose attribution to non-human beings is problematic. In Switzerland, the solution has been to equate respect for the dignity of non-human beings, particularly animals, with taking their interests seriously, for those who have interests. In short: Put all the existing interests in a balance and decide in favor of the most dominant (ECNH and FCAE 2001).

## The Abuse of Dignity?

Dignity is a concept that is used in a great diversity of contexts, and in each context, appeals to human dignity abound; their frequency is manifestly increasing. Is it justified? In this last section, I will try to answer this question, asking first if we *could* dispense with the concept and second if we *should* dispense with it.

Could we dispense with the concept of “dignity”? Obviously, we could. Since “dignity” is synonymous with “intrinsic value,” we could simply replace the first expression by the second. Macklin makes another proposition, to speak of autonomy instead of dignity. But this proposition could be accepted only if respecting the intrinsic value of persons is equivalent to respecting their autonomy. If this were the case, then voluntary slavery or dire poverty would not per se constitute infringements of human dignity, a conclusion that only some libertarians would endorse. Perhaps, therefore, we could supplement autonomy with basic rights. This will satisfy liberals and, probably, most jurists. Of course, conservatives and perfectionists, that is people who think that respect for the human person is not exhausted by respect for individual rights, will not agree.

There is another problem with this strategy of replacement: It is no less efficacious against rights than it is against dignity. We could (and should) dispense with rights, say some authors. Bentham and Marx are two defenders of this position. Actually, from a conceptual point of view, the argument against dignity can be used against rights. In principle, rights could be dispensed with and replaced by concepts such as “happiness,” “good,” or “value.” Thus, the ethical work can be done without rights, which would possess only rhetorical force (Baertschi 2008). However, this critique, valid as it is, is not necessarily fatal. Concerning rights, Loren Lomasky concedes the conceptual point. But for him rights are, nevertheless, important for our morality, since rhetoric is the art of putting something—here, certain values—in a prominent place: “The very vigor and insistence of rights advocates may lead us to conjecture that the language of right has an importance which would not survive a shift of idiom” (Lomasky 1987, 10). Could the same claim be made for dignity?^12^

This question leads us to another (the second part of our issue): Should we dispense with the concept of “dignity”? The answer is affirmative only if we cannot give an answer in Lomansky’s guise. In other words, can we propose an argument in favor of dignity that is similar to that in favor of rights? If not, dignity will be a useless concept; if so, it will be a useful one. In my opinion, we are in possession of such an argument: Dignity is useful in order to cast a full light on certain practices that we do not want established—or re-established, for instance practices resembling slavery and torture. It is in order to denounce such degrading treatments that, in modern and contemporary times, we appeal to human dignity, because we think that it is insufficient to invoke rights or the mere intrinsic value of human beings. In this context, it is morally necessary to use another word—even a traditional one—because of the importance of the values placed in jeopardy and of the moral agenda of what we hope will lead to moral progress. Therefore, it is not justified to speak of the “stupidity of dignity.” Pinker would agree with much of this, since he claims:

Dignity is a phenomenon of human perception. … Certain features in another human being trigger ascriptions of worth. … The perception of dignity in turn elicits a response in the perceiver. … The appearance of dignity triggers a desire to esteem and respect the dignified person. This explains why dignity is morally significant: We should not ignore a phenomenon that causes one person to respect the rights and interests of another (2008, 7).

However, to extend the application of dignity, as conservatives do, is to diminish its strength and to lose the widespread consensus respect for dignity possesses in the context of degrading treatments. Sometimes, dignity is even invoked in bioethical debates to conceal a bad argument or the absence of an argument. Unfortunately, this is not the only term used when the parties are short of rational considerations; think of expressions such as “But this is against human rights!” or “But that is eugenics!” Like dignity, human rights and eugenics are very often used in bad rhetoric.

Bad rhetoric apart, we can nevertheless understand why “dignity” can be rather easily extended. There is a conceptual reason we already know: In itself, dignity is simply a name for the intrinsic value of a being, and its meaning can only be determined by the conception we entertain of the nature of this being (i.e., its main valuable characteristics). If we entertain a perfectionist and rich notion of these characteristics, if we have a thick conception of the good life for a person, dignity could be put forward in opposition to an intervention that has received informed consent and does no wrong to any third party. In this sense, Pinker is right when he states: “The concept of dignity is natural ground on which to build an obstructionist bioethics” (2008, ¶18). The appeal to dignity is, then, only a way to oppose a liberal stance; it does not justify anything by itself.

The content of “dignity” must be determined and is determinable only by valuable features of the person, features that now as before refer to an aspect of rationality. We can fill it with a conservative conception, but there is nothing necessary in this respect. It is also possible to fill it with liberal content, even with libertarian content; for instance, by adopting a conception of dignity grounded in the notion of autonomy.

A person is distinctive because of certain rational properties. Let us accept as a possible interpretation of this thesis that autonomy—the capacity to direct one’s life—is one of these relevant capacities, even the most important. It would follow that human dignity is grounded in the capacity for autonomy.^13^ From this point of view, it would not be possible to appeal to human dignity to obstruct a procedure that has received informed consent and does no wrong to anybody else. Such an obstruction would disrespect dignity. As Adam Schulman asks:

If the rational will alone is the seat of human dignity, why should it matter if we are born of cloned embryos, or if we enhance our muscles and control our moods with drugs, or if we sell our organs on the open market? (2008, 11).

We could even extend such a conception to transhumanism: In voluntarily “rectifying the flaws in our design” (Rubin 2008, 157), we complete and supplement our nature with a beneficial use of our rational powers.

Violations of dignity are always a debasement of moral status, but there are several ways of understanding this depending on how one conceives what is appropriate for a being with the status of a person. In terms of liberal thought, instrumentalization is an example of such debasement; for perfectionists, the list is much longer. But is it not an open moral question to determine if everything freely consented to is by that fact morally good or permissible or praiseworthy? To condemn the use of dignity as a stupidity should not be used as another rhetorical weapon, even in favor of the liberal side of the debate. Liberals *and* conservatives ought to offer arguments.

All these uses and abuses are made possible because “dignity”—like “person,” “moral status,” and “intrinsic value”—has little substantial content in itself. It is a kind of *thin* evaluative term, and as such, it becomes easily pluralistic when it is given more content. Different conceptions of what a human being is and of its intrinsic value (i.e., of its dignity) are at play and in opposition, each with its own moral demands. As Schulman states: “A variety of strong convictions can be derived from powerful but conflicting intuitions about what human dignity demands of us” (2008, 5).

Debates must acknowledge this and build arguments for and against these conceptions, without waving “flag words” like “dignity” or “stupidity.” Therefore, the question asked by Alasdair Cochrane is appropriate: “The dignity of human beings tells us that we have certain important obligations towards them. But which obligations?” (2010, 4). It would nevertheless be a mistake to believe that the task of answering this question should fall on the concept of dignity. To be sure, if this concept is thin, it is not hollow. Yet the most it can tell us is that it grounds obligations we have toward beings possessing *rational* capacities because of these capacities, forbidding us from treating them like non-rational beings: that is, without respecting their own true goals.

## Conclusion

Dignity is a very old concept, but its uses in bioethical debates are rather new. It has rapidly acquired a rhetorical overtone, especially for authors who do not think that considerations of rights exhaust the ethical debate, like various perfectionists such as conservatives and religious thinkers. It is, however, quite possible to use it in a liberal spirit or, more broadly, in a spirit that places autonomy and liberty at the fore. I have tried to show that this concept, *if used in conformity with its traditional formal meaning*, as synonymous with intrinsic value and as an indicator of the importance of the moral status of personhood, does not constitute a problem and cannot be hijacked by a party.

Of course, it is possible that, in the future and contrary to our moral tradition, dignity and personhood will separate and the debates surrounding dignity will be defined along the lines of Pinker’s critique. This may be the case if the American controversy prevails and will represent bad news for bioethics. In my opinion, the best way to prevent such an issue from arising is to restrict the concept of dignity to its traditional and consensual use. In other words, that human beings or persons possess an intrinsic value that demands to be respected *in the context of condemnation of degrading practices* resembling slavery and torture; dignity ought to be used “as a placeholder against great evils” (Neuhaus 2008, 218). To do that, the language of rights appears to be a little too lightweight.

## Footnotes

1 My aim is not to provide a thorough piece of historical analysis. If some features of Kant’s and Aquinas’ conceptions are analyzed, it is only because they are representative of the conceptual structure in which I am interested.

2 See Baertschi (2005, 168–169). Lennart Nordenfelt (2004, 69) has identified three kinds of dignity: dignity of moral stature, dignity of identity, and human dignity (or *Menschenwürde*). However, I diverge somewhat from him on dignity of identity.

3 Aquinas, one of the first authors to make sense of the concept of human dignity, claims that this dignity can be lost (1947, IIa–IIae, q. 64, a. 2, ad 3).

4 Kant speaks of dignity as an *internal* value, but “internal” is synonymous with “intrinsic.”

5 One could object that “dignity” presupposes an essentialist conception of human beings and that such a conception, like any essentialism, is at best controversial. I agree that essentialist conceptions are fraught with difficulties, but their discussion is beyond the scope of this paper.

6 This thesis has been defended by G.E. Moore. See Rabinowicz and Rønnow-Rasmussen 2000; Baertschi 2012.

7 “Ce qui fait différer l’homme d’avec la bête—Ce n’est pas l’estomac ni le pied ni la tête—La face ni les yeux; c’est la seule raison” (Ronsard 1989, 236).

8 A third founding father of Western ethics, John Locke, is of the same mind (Locke 1690, 540).

9 I have discussed elsewhere the question of the embryo’s moral status in an individualist spirit (Baertschi and Mauron 2010).

10 I owe this objection to an anonymous reviewer.

11 For Germany, another country that gives a prominent place to dignity in its Constitution, see Weithman (2008).

12 The use of the expression “rhetoric” here should not be misinterpreted. It does not amount to the derogatory thesis that talk of dignity or rights would only be rhetoric (and not ethics). In using this expression, I intend to stress the function of rhetoric in our moral and social life—a very important function indeed.

13 Significantly, this conception would even be more germane to tradition than the conservative one, as the latter supplements the concept of a person with non-rational elements (Kass 2005).
